# Turkish health system reform from the people perspective

**DOI:** 10.1186/1471-2458-14-S1-P14

**Published:** 2014-01-29

**Authors:** Saad A Ali Jadoo Alazawi, Syed Aljunid, Seher N Sulku, Amrizal M Nur

**Affiliations:** 1 United Nations University, International Institute for Global Health (UNU-IIGH), 56000 Cheras, Kuala Lumpur, Malaysia; 2 Department of Econometrics, Economics and Management Sciences Faculty, Gazi University, Ankara, Turkey

## Background

A cross sectional study was carried out in Turkey to assess the consequences of the health system reforms that had been carried out in the last decade.

## Materials and methods

A total of 482 heads of household have been selected by using a multi-stage sampling method. Data was collected via household’s structured questionnaire. The response rate was 71.7% and data was analysed by using SPSS version 16.0. Age range of respondents was 28 to 73 years old with the mean age of 46.60 ±11.85 years. The majority of clients were married (59.8%) and were employed at the time of data collection (87.2%).

## Results

Among the respondents, more than two thirds (69.3%) of the population believed that the changes have had a positive effect on the accessibility (82.0%); availability of resources (74.9%); quality of care (73.2%) and attitude by politician/mass media (76.1%). The elderly, married female, and those who believed that people are happier than 10 years ago have a more positive view of the changes, while the unemployed, low educated and those who perceived themselves as unhealthy showed less positive views. At the same time, the majority of respondents (77.6%) preferred the current health care system than the past.

## Conclusions

This study could bring closer the viewpoints of different categories of people and officials of the health system reforms in a way that could lead to the better implementation of such reforms.

